# Supplementary material for: Finding a Husband: Using Explainable AI to Define Male Mosquito Flight Differences
Source: Biology (Basel). 2023 Mar 24;12(4):496. doi: 10.3390/biology12040496 (PMC10135534; doi:10.3390/biology12040496)

Finding a Husband: Using Explainable AI to Define Male Mosquito Flight Differences

Yasser M. Qureshi [1,*], Vitaly Voloshin [1,2], Luca Facchinelli [3], Philip J. McCall [3], Olga Chervova [4], Cathy E. Towers [1], James A. Covington [1] and David P. Towers [1]

[1]School of Engineering, University of Warwick, Coventry, CV4 7AL, UK

[2]Royal Botanical Garden, Kew, Richmond, London, TW9 3AE, UK

[3]Liverpool School of Tropical Medicine, Pembroke Place, Liverpool, L3 5QA, UK

[4]UCL Cancer Institute, University College London, London, WC1E 6DD, UK

* Correspondence: yasser.qureshi@warwick.ac.uk

Table S1. Equations for Each Evaluation Metric. Where TN, TP, FN, and FP denote the number of true negatives, true positives, false negatives and false positives respectively.

Metric Name	Equation
Accuracy	$\frac{TN+TP}{TN+FN+TP+FP}$
Balanced Accuracy	$\frac{1}{2}\left(\frac{TP}{TP+FN}+\frac{TN}{TN+FP}\right)$
Recall Score	$\frac{TP}{TP+FN}$
Precision Score	$\frac{TP}{TP+FP}$
F1 Score	$2\cdot\frac{\text{precision}\cdot\text{recall}}{\text{precision}+\text{recall}}$

Figure S1. Track duration against displacement coloured by each trial with 95% Confidence Interval.

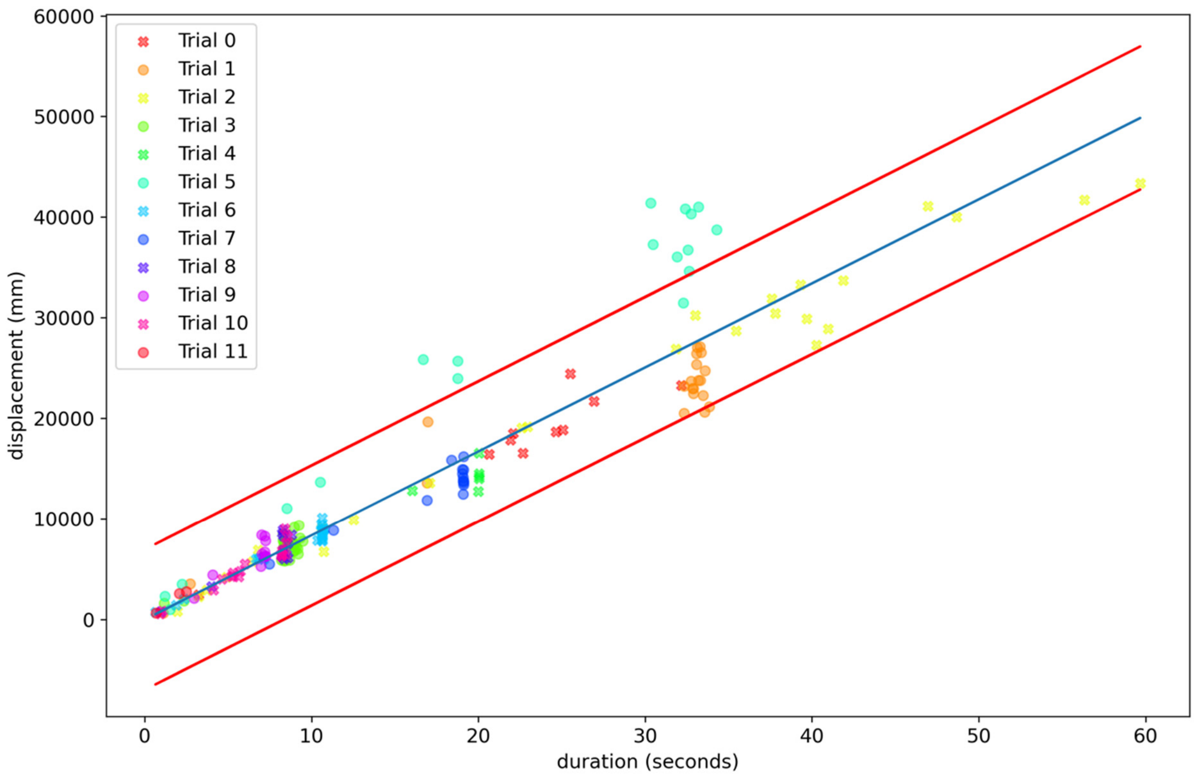

Table S2. Classification Performance using Trial recorded in high wind speeds

	Train set	Test set (Trial in high wind speed)	Test set (Without trial in high wind speed)
**Accuracy**	0.854 (0.774 - 0.930)	0.126 (0.000 - 0.243)	0.756 (0.334 - 0.925)

Table S3. Features extracted and their corresponding statistical features from mosquito tracks

Feature Names	Statistical Properties	
Absolute Velocity	- Mean<br>- Median<br>- Standard Deviation<br>- 1st Quartile<br>- 3rd Quartile<br>- Kurtosis	- Skewness<br>- Number of local minima<br>- Number of local maxima<br>- Number of zero crossings
Absolute Acceleration	- Mean<br>- Median<br>- Standard Deviation	- Skewness<br>- Number of local minima<br>- Number of local maxima

	- 1st Quartile<br>- 3rd Quartile<br>- Kurtosis	- Number of zero crossings
Absolute Jerk	- Mean<br>- Median<br>- Standard Deviation<br>- 1st Quartile<br>- 3rd Quartile<br>- Kurtosis	- Skewness<br>- Number of local minima<br>- Number of local maxima<br>- Number of zero crossings
X Velocity	- Mean<br>- Median<br>- Standard Deviation<br>- 1st Quartile<br>- 3rd Quartile<br>- Kurtosis	- Skewness<br>- Number of local minima<br>- Number of local maxima<br>- Number of zero crossings
Y Velocity	- Mean<br>- Median<br>- Standard Deviation<br>- 1st Quartile<br>- 3rd Quartile<br>- Kurtosis	- Skewness<br>- Number of local minima<br>- Number of local maxima<br>- Number of zero crossings
Z Velocity	- Mean<br>- Median<br>- Standard Deviation<br>- 1st Quartile<br>- 3rd Quartile<br>- Kurtosis	- Skewness<br>- Number of local minima<br>- Number of local maxima<br>- Number of zero crossings
X Acceleration	- Mean	- Skewness

	- Median<br>- Standard Deviation<br>- 1st Quartile<br>- 3rd Quartile<br>- Kurtosis	- Number of local minima<br>- Number of local maxima<br>- Number of zero crossings
Y Acceleration	- Mean<br>- Median<br>- Standard Deviation<br>- 1st Quartile<br>- 3rd Quartile<br>- Kurtosis	- Skewness<br>- Number of local minima<br>- Number of local maxima<br>- Number of zero crossings
Z Acceleration	- Mean<br>- Median<br>- Standard Deviation<br>- 1st Quartile<br>- 3rd Quartile<br>- Kurtosis	- Skewness<br>- Entropy<br>- Number of local minima<br>- Number of local maxima<br>- Number of zero crossings
Angular Velocity (3D, X-Y, Y-Z, Z-X)	- Mean<br>- Median<br>- Standard Deviation<br>- 1st Quartile<br>- 3rd Quartile<br>- Kurtosis	- Skewness<br>- Number of local minima<br>- Number of local maxima<br>- Number of zero crossings
Angular Acceleration (3D, X-Y, Y-Z, Z-X)	- Mean<br>- Median<br>- Standard Deviation<br>- 1st Quartile<br>- 3rd Quartile	- Skewness<br>- Number of local minima<br>- Number of local maxima<br>- Number of zero crossings

	- Kurtosis
Angle of Flight	- Mean<br>- Median<br>- Standard Deviation<br>- 1st Quartile<br>- 3rd Quartile<br>- Kurtosis<br>- Skewness<br>- Number of local minima<br>- Number of local maxima<br>- Number of zero crossings
Centroid Distance Function	- Mean<br>- Median<br>- Standard Deviation<br>- 1st Quartile<br>- 3rd Quartile<br>- Kurtosis<br>- Skewness<br>- Number of local minima<br>- Number of local maxima<br>- Number of zero crossings
Orthogonal Components (Persistence velocity, turning velocity and inclination velocity)	- Mean<br>- Median<br>- Standard Deviation<br>- 1st Quartile<br>- 3rd Quartile<br>- Kurtosis<br>- Skewness<br>- Number of local minima<br>- Number of local maxima<br>- Number of zero crossings
Straightness	
Convex Hull	- Volume<br>- Surface area
Curvature Scale Space	- Mean<br>- Standard Deviation
Fractal Dimension	
Curvature (X-Y, Y-Z, Z-X)	- Mean<br>- Standard Deviation

Table S4. Features selected from feature selection process

Feature Names
Angle of Flight (1st quartile)
Angle of Flight (3rd quartile)
Angle of Flight (median)
Angle of Flight (no of local maxima)
Angle of Flight (standard deviation)
Angular Acceleration (1st quartile)
Angular Acceleration (3rd quartile)
Angular Acceleration X-Y (1st quartile)
Angular Acceleration X-Y (3rd quartile)
Angular Acceleration X-Y (no of local maxima)
Angular Acceleration X-Y (no of zero-crossings)
Angular Acceleration X-Y (standard deviation)
Angular Acceleration X-Z (1st quartile)
Angular Acceleration X-Z (3rd quartile)
Angular Acceleration X-Z (kurtosis)
Angular Acceleration X-Z (standard deviation)
Angular Acceleration Y-Z (1st quartile)
Angular Acceleration Y-Z (3rd quartile)
Angular Acceleration Y-Z (no of local maxima)
Angular Acceleration Y-Z (no of zero-crossings)
Angular Acceleration Y-Z (standard deviation)
Angular Velocity X-Y (1st quartile)
Angular Velocity X-Y (median)
Angular Velocity X-Z (3rd quartile)
Angular Velocity X-Z (median)
Angular Velocity Y-Z (3rd quartile)
Centroid Distance Function (1st quartile)
Centroid Distance Function (no of local maxima)
Centroid Distance Function (no of local minima)
Curvature Scale Space (mean)
Curvature X-Y (standard deviation)
Curvature X-Z (standard deviation)
Curvature Y-Z (standard deviation)
Fractal Dimension
Inclination Velocity (3rd quartile)
Inclination Velocity (kurtosis)
Inclination Velocity (no of local maxima)
Inclination Velocity (skewness)
Inclination Velocity (standard deviation)
Persistence Velocity (no of local maxima)
Persistence Velocity (no of local minima)

Persistence Velocity (no of zero-crossings)
Radial Acceleration (1st quartile)
Radial Acceleration (3rd quartile)
Radial Acceleration (no of local maxima)
Radial Acceleration (no of local minima)
Radial Acceleration (no of zero-crossings)
Radial Jerk (1st quartile)
Radial Jerk (3rd quartile)
Straightness
Turning Velocity (3rd quartile)
Turning Velocity (no of local maxima)
Turning Velocity (no of zero-crossings)
Turning Velocity (skewness)
Turning Velocity (standard deviation)
X Acceleration (1st quartile)
X Acceleration (3rd quartile)
X Velocity (1st quartile)
X Velocity (3rd quartile)
X Velocity (no of local maxima)
X Velocity (no of local minima)
X Velocity (standard deviation)
Y Acceleration (1st quartile)
Y Acceleration (kurtosis)
Y Acceleration (no of local maxima)
Y Acceleration (no of local minima)
Y Acceleration (no of zero-crossings)
Y Velocity (1st quartile)
Y Velocity (kurtosis)
Y Velocity (no of local maxima)
Y Velocity (skewness)
Z Acceleration (1st quartile)
Z Acceleration (no of zero-crossings)
Z Velocity (1st quartile)
Z Velocity (3rd quartile)
Z Velocity (kurtosis)
Z Velocity (no of local maxima)

Table S5. Classification Performance using Duration and Distance travelled as features.

	Balanced Accuracy	Precision	Recall	F1 Score	ROC AUC Score	PR AUC Score
**Test set (complete set)**	0.801 (0.630-0.896)	0.222 (0.120-0.303)	0.719 (0.366-0.919)	0.336 (0.189-0.452)	0.843 (0.630-0.942)	0.979 (0.924-0.997)

Figure S2. Boxplot of velocities of each track in male trials. Trial 5 was conducted on the 28th Aug 2010.

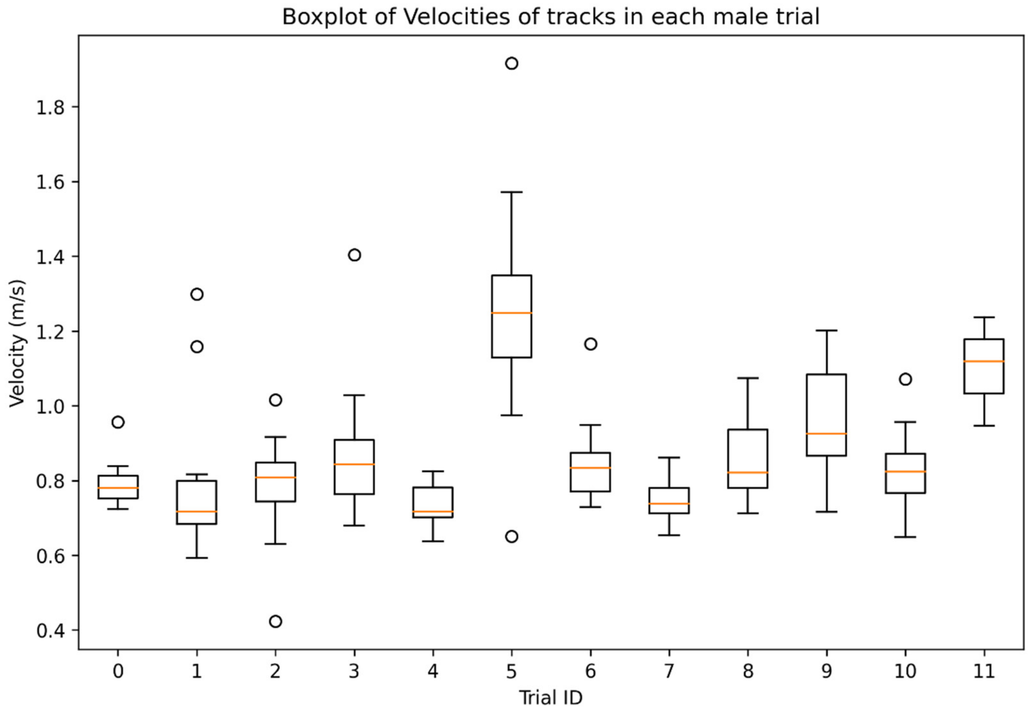

Figure S3. Boxplot of distance travelled by track in each male trials.

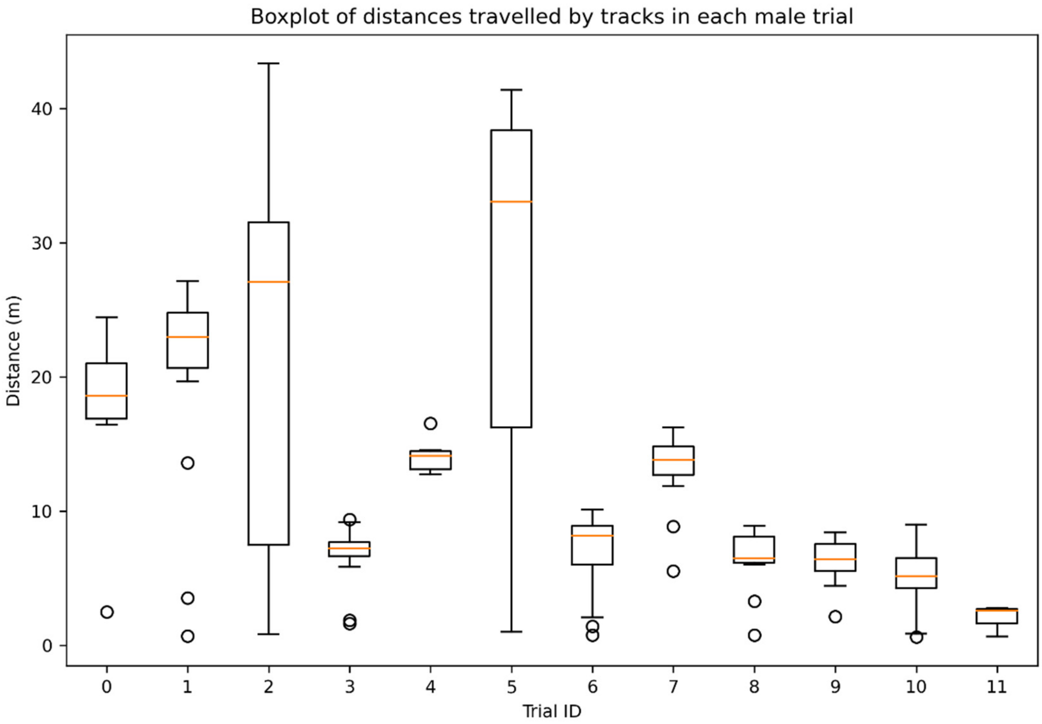

Figure S4. Boxplot of durations of track in each male trials.

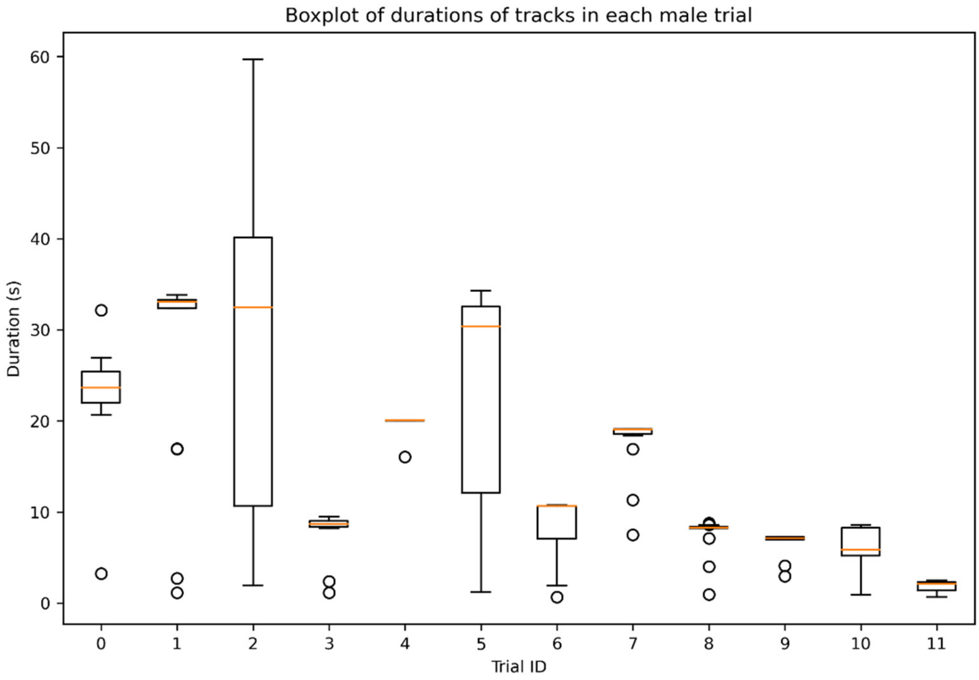

Figure S5. Number of segments per trial.

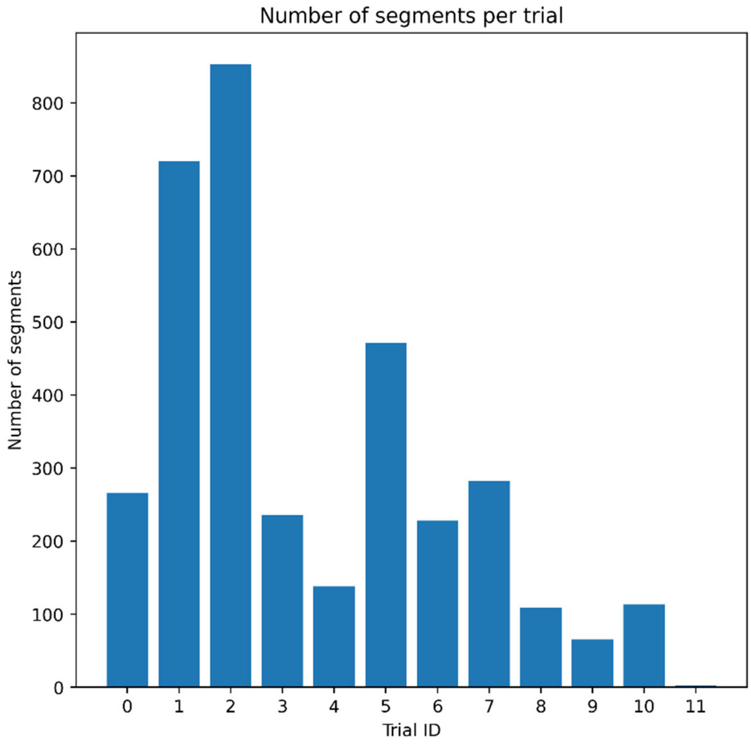

Figure S6. Number of tracks per trial.

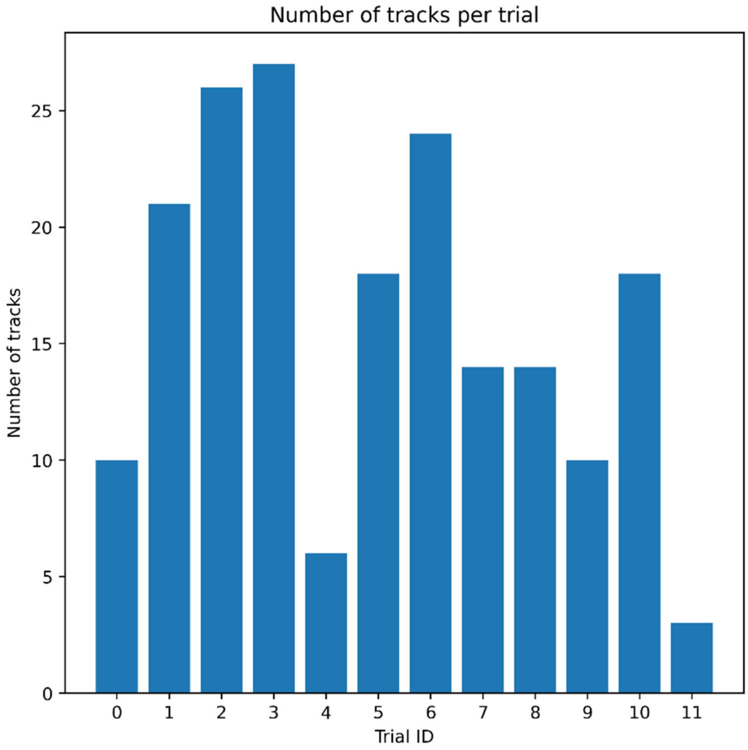

Figure S7. Normalised histogram of Velocity.

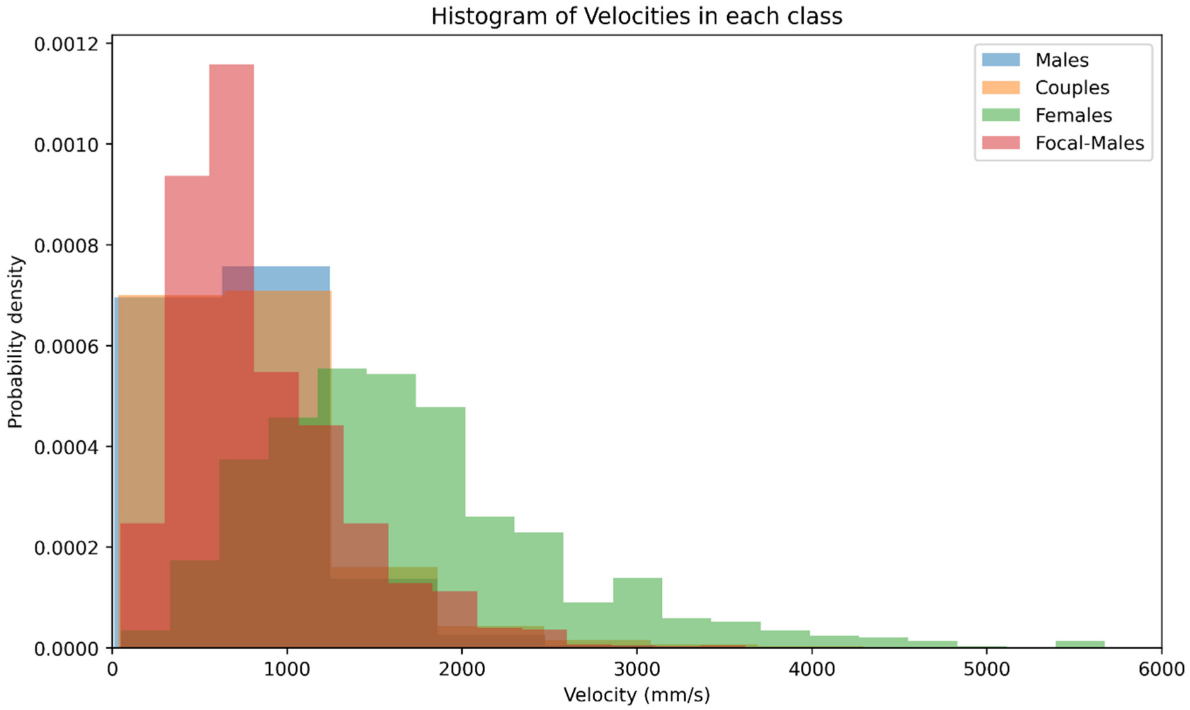

Figure S8. Normalised histogram of Acceleration.

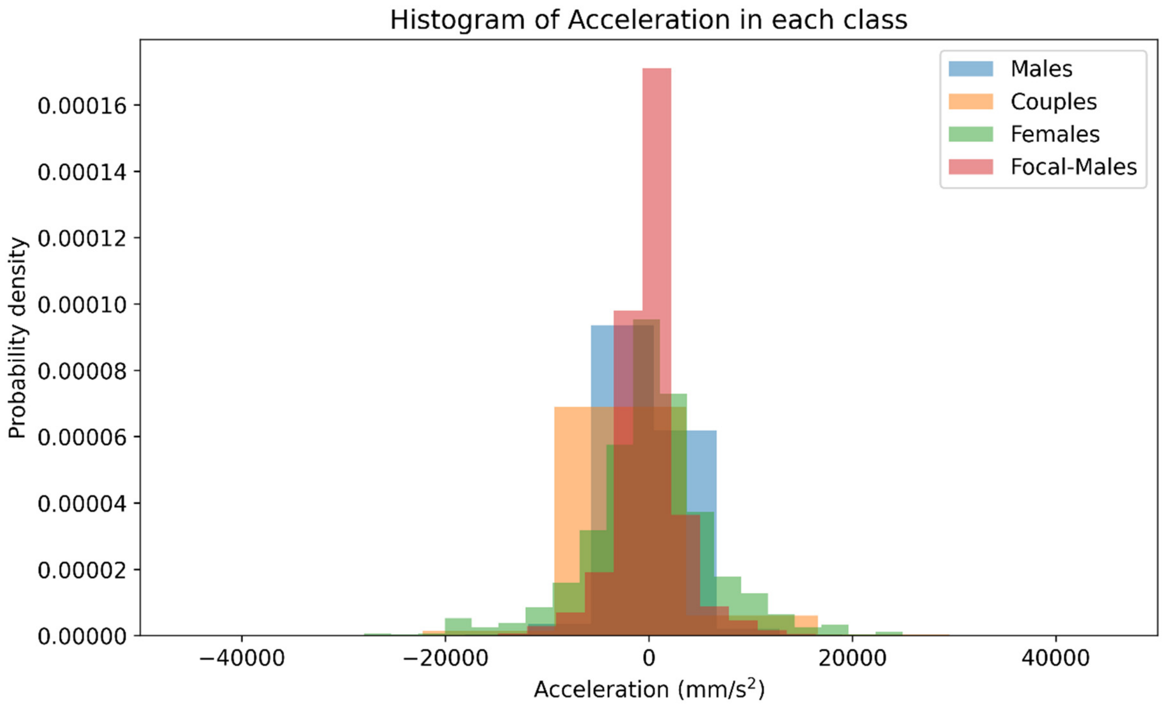

Table S6. Classification performance without trials 8, 9 and 11.

	Accuracy
**Train set (Male only)**	0.779 (0.746 - 0.838)
**Test set (Male only)**	0.609 (0.142 - 0.859)
**Test set (Couple only)**	0.636 (0.589 - 0.699)
**Test set (Female only)**	1.000 (1.000 - 1.000)
**Test set (Focal Male)**	0.800 (0.667 - 0.833)

	Balanced Accuracy	ROC AUC Score
**Test set (complete set)**	0.648 (0.453 - 0.752)	0.690 (0.582 - 0.730)

	PR AUC Score	Precision	Recall	F1 Score
**Male**	0.394 (0.273 - 0.461)	0.451 (0.199 - 0.575)	0.640 (0.250 - 0.856)	0.526 (0.222 - 0.68)
**Non-Male**	0.868 (0.851 - 0.890)	0.811 (0.717 - 0.900)	0.656 (0.612 - 0.715)	0.723 (0.686 - 0.752)
**Overall**	0.631 (0.563 - 0.666)	0.631 (0.458 - 0.729)	0.648 (0.453 - 0.752)	0.625 (0.454 - 0.716)

Figure S9. SHAP summary plot displaying all features for the best fold.

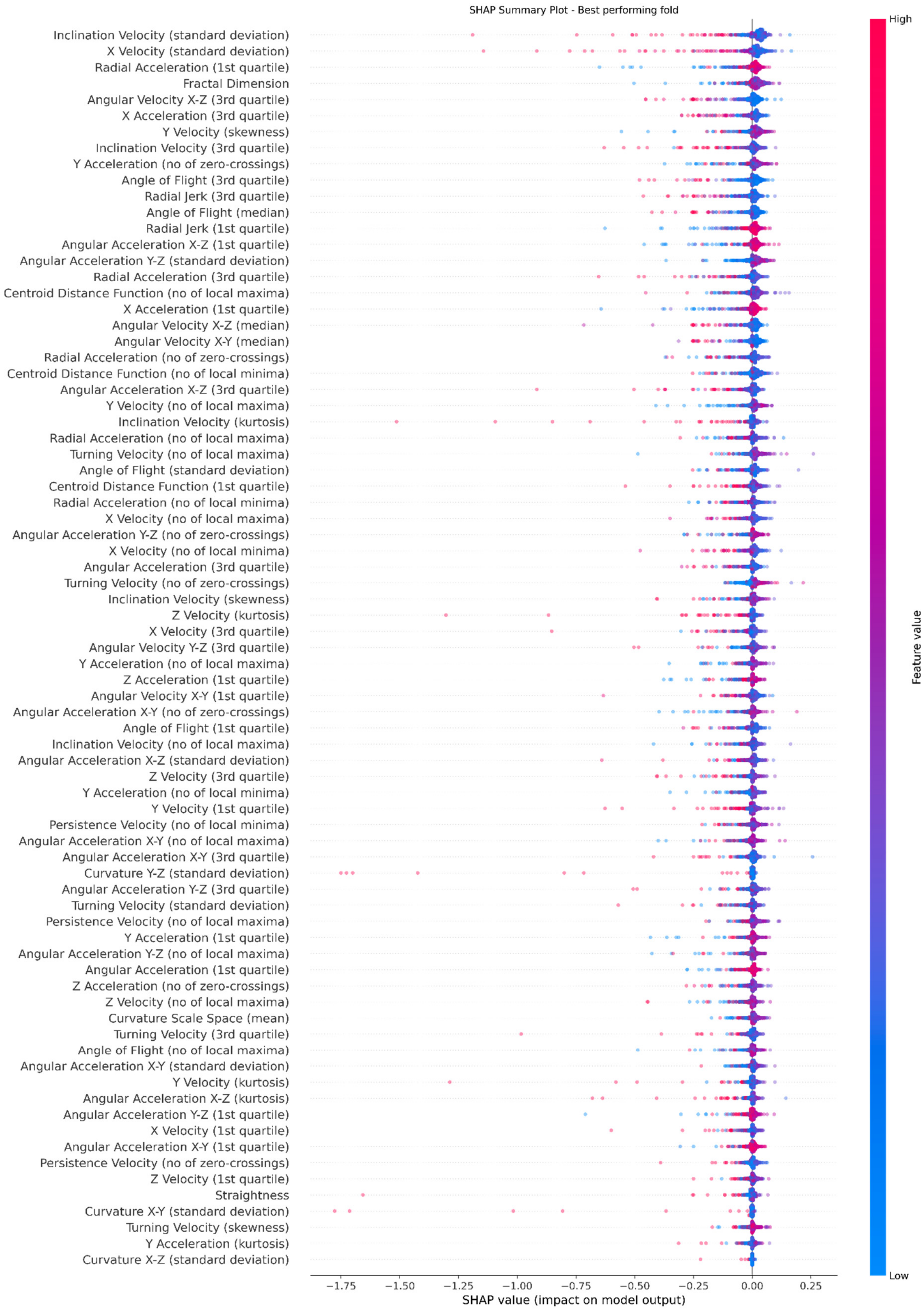

Figure S10. SHAP summary plot displaying all features for the worst fold.

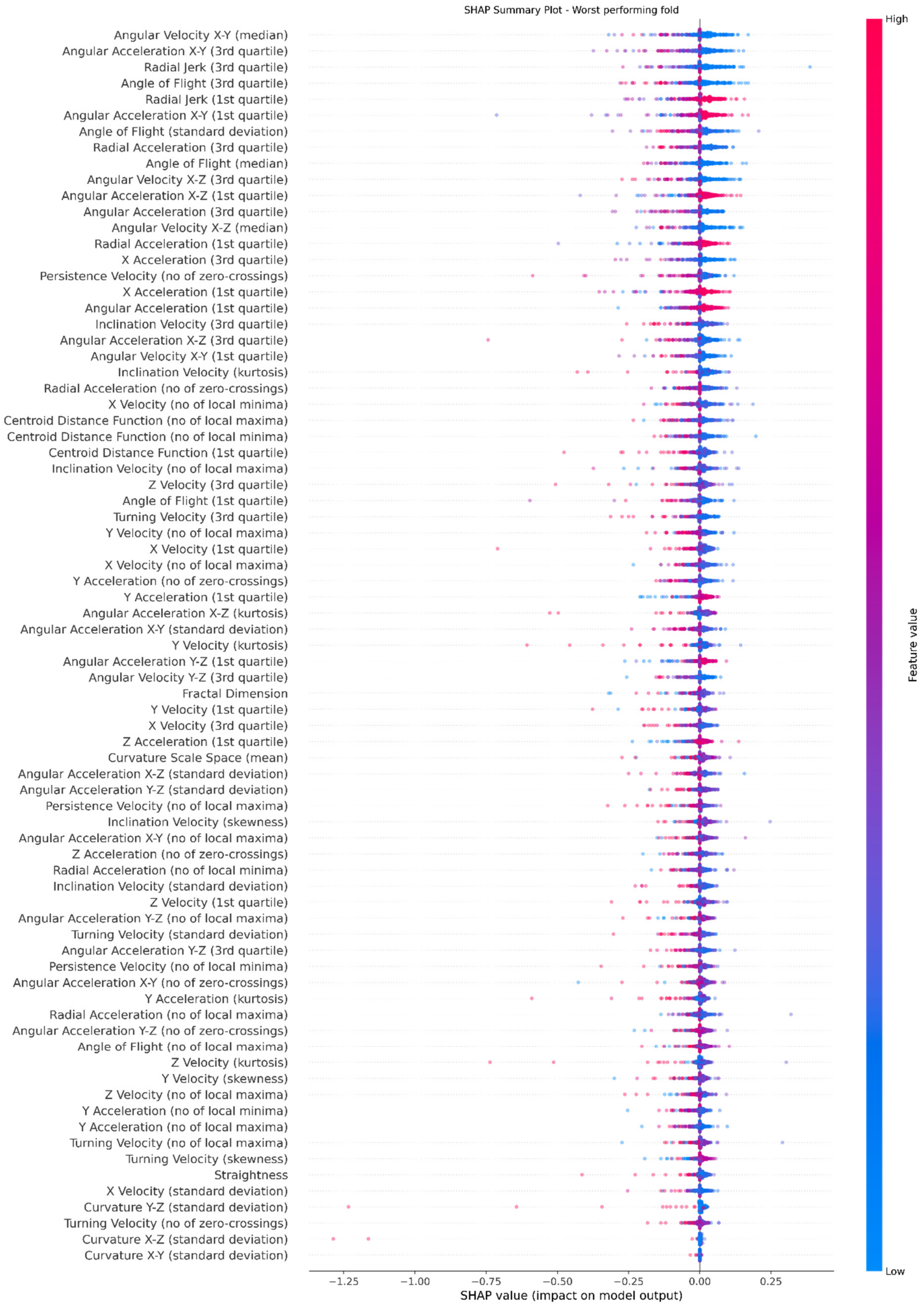

Figure S11. SHAP bar plot displaying all features for the best fold.

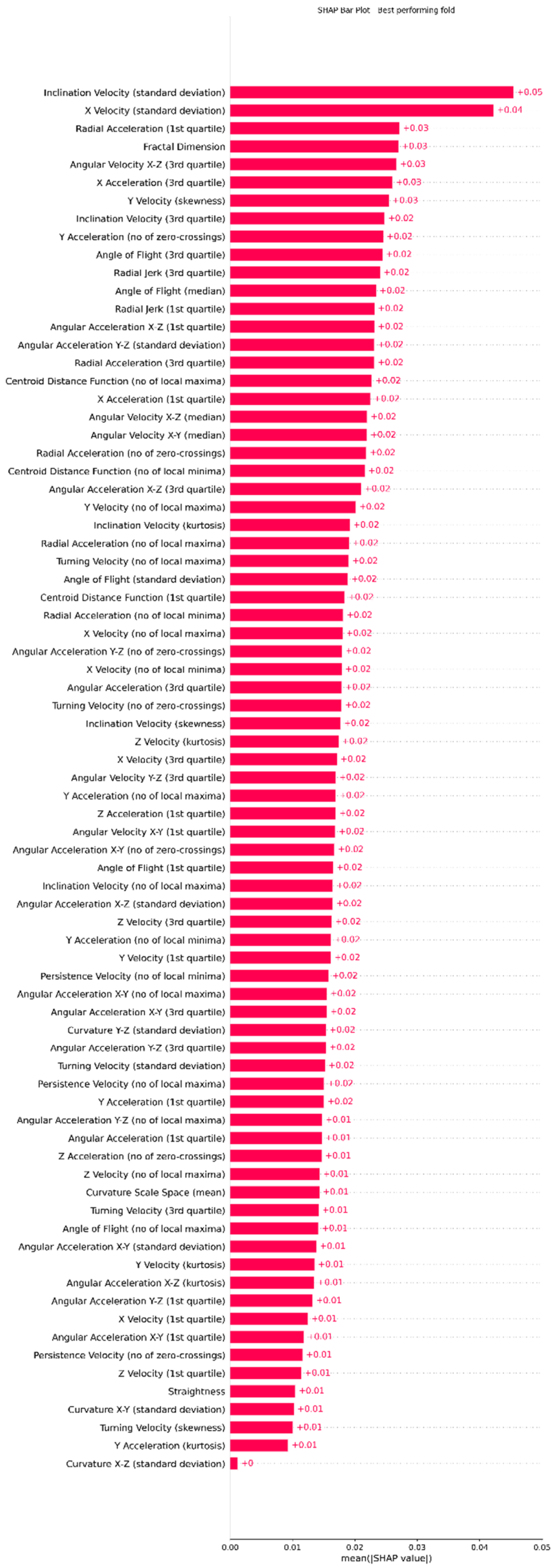

Figure S12. SHAP bar plot displaying all features for the worst fold.

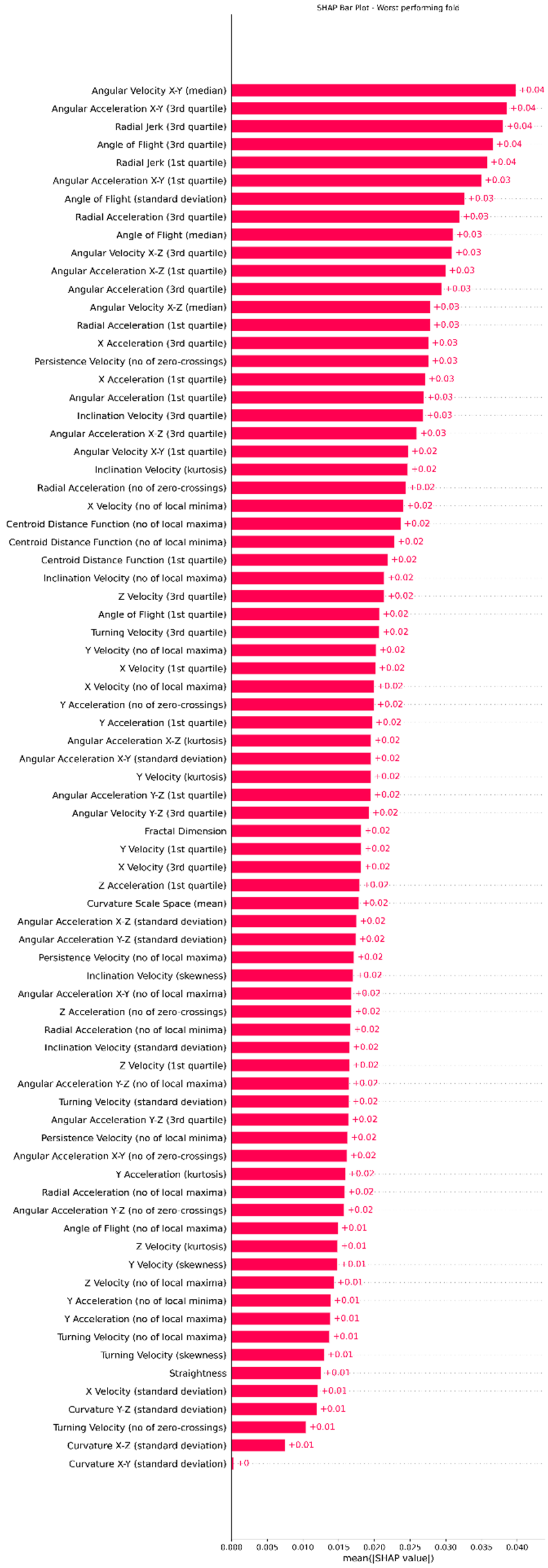

Figure S13. Example of a Scatter plot of Angular Acceleration (1st quartile)

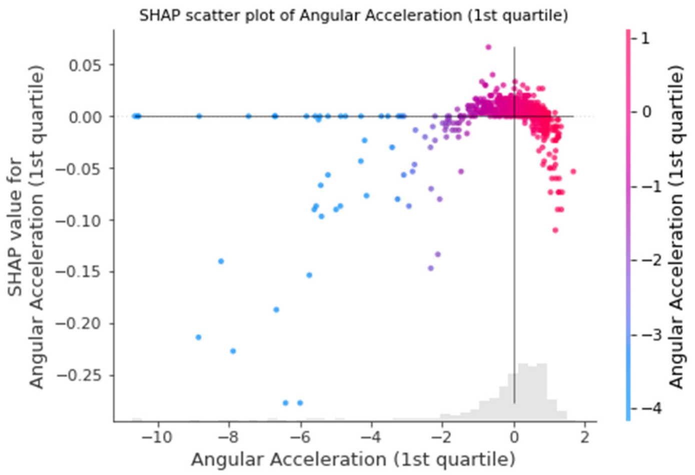

Supplement: Supplementary file 1 [file biology-12-00496-s001.zip › biology-2241419-supplementary.pdf]
